# Pathomics-based machine learning model predicts interferon-gamma expression in head and neck squamous cell carcinoma patients

**DOI:** 10.3389/fimmu.2025.1652003

**Published:** 2025-11-24

**Authors:** Jintao Yu, Wei Teng, Gang Yu, Zheng Wang, Bin Bai, Yan Wang, Fei Wang

**Affiliations:** 1 Department of Otolaryngology, The First Hospital of China Medical University, Shenyang, China; 2 Second Department of Infectious Diseases, The First Hospital of China Medical University, Shenyang, China; 3 Department of Dermatology, The First Hospital of China Medical University, Shenyang, China; 4 Key Laboratory of Immunodermatology, Ministry of Education, and National Health Commission; National Joint Engineering Research Center for Theranostics of Immunological Skin Diseases, Shenyang, China

**Keywords:** biomarkers, IFNg, machine learning, head and neck squamous cell carcinoma, pathomics

## Abstract

**Introduction:**

Interferon-gamma (IFNG) plays a key role in immune responses in head and neck squamous cell carcinoma (HNSCC) and impacts the effectiveness of immune checkpoint inhibitors. This study developed a machine learning model that leverages pathomics to forecast IFNG expression based on histopathological images while thoroughly examining the tumor immune microenvironment in HNSCC.

**Methods:**

The analysis involved 271 cases from The Cancer Genome Atlas (TCGA)-HNSCC, with validation using 71 patients from the Hospital. Significant links were found between IFNG expression, clinical features, and survival outcomes. For histopathological image processing, tumor regions were segmented using the OTSU algorithm, and 1,488 features were extracted with PyRadiomics. A feature selection strategy that integrated minimum Redundancy Maximum Relevance (mRMR) and Recursive Feature Elimination (RFE) pinpointed 30 essential features, which were then employed to construct a Gradient Boosting Machine (GBM) predictive.

**Results:**

This model demonstrated strong performance in predicting survival, with AUC values of 0.836 in the TCGA training set, 0.753 in the validation set, and 0.740 in the hospital dataset. Gene set variation analysis revealed distinct pathway activation patterns among PS subgroups, indicating that pathways associated with immune evasion were more prominent in patients with high PS. Survival analysis substantiated that patients exhibiting elevated IFNG expression experienced a longer median survival.

**Discussion:**

In conclusion, the study shows that pathomics from histopathological images can predict IFNG expression. The correlation between IFNG and pathomics provides a valuable biomarker framework for elucidating the pathophysiology of HNSCC and may inform personalized therapeutic strategies through non-invasive characterization of the immune microenvironment.

## Introduction

Head and neck squamous cell carcinoma (HNSCC) is a serious condition that poses a significant threat to global health, with over 650,000 new cases and approximately 330,000 deaths reported annually (1, 2). Although advancements have been made in treatment methods such as surgery, radiation, and chemotherapy, the five-year survival rate for patients diagnosed with HNSCC remains disappointingly low, hovering around 50-60% (3). To tackle these pressing issues, it is essential to gain a deeper understanding of the biological factors that contribute to HNSCC. Furthermore, the disease's inherent heterogeneity and aggressive characteristics significantly impact the development of treatment strategies, highlighting the urgent need for improved prognostic markers and tailored treatment approaches that can enhance patient outcomes (4).

The clinical management of HNSCC is challenged by the disease's complexity and the limitations of current diagnostic and prognostic tools. Traditional staging systems often fail to capture the full extent of intertumoral heterogeneity present in HNSCC tumors, resulting in less effective treatment planning and patient stratification (5). Therefore, integrating molecular and pathomics data is essential for refining prognostic assessments and guiding precision oncology efforts.

The Interferon-gamma (*IFNG*) gene encodes IFN-γ, a cytokine that plays a pivotal role in immune responses to tumors (6). IFN-γ is crucial for activating immune cells, enhancing antigen presentation, and promoting anti-tumor immunity. In various cancers, increased levels of IFN-γ signaling, either in tumor tissue or peripheral blood, are associated with enhanced immune activity and are significantly correlated with longer overall survival (OS), progression-free survival (PFS), and improved response rates to immune checkpoint inhibitors (ICBs) (7). Previous studies have demonstrated that in HPV-positive HNSCC, tumor tissue is notably enriched with CD8^+^ IFN-γ^+^ cells, which are accompanied by elevated immune checkpoint markers such as PD-1 and LAG-3 (8, 9). This suggests the presence of a sustained, yet not exhausted, T cell response, and patients with this immune profile have a better 5-year OS compared to HPV-negative counterparts. Moreover, a clinical trial found that IFN-γ signaling genes were significantly higher in biopsy samples from patients with metastatic/recurrent HNSCC who responded well to pembrolizumab, compared to non-responders (10). Given the essential role of IFN-γ in tumor immunity, it is increasingly being explored as a potential biomarker for guiding immune-based therapies. However, traditional methods for measuring IFN-γ, such as immunohistochemistry (IHC), multiplex IHC, or ELISA, have several limitations. These methods can suffer from variability depending on the sampling site, handling conditions, and antibody sensitivity.

Pathomics Score (PS) is a predictive score developed using machine learning techniques based on histopathological features (11). By utilizing a pathological Gradient Boosting Machine (GBM) model, this study aims to explore the correlation between PS and IFNG expression, and to validate their consistency in predicting prognosis, providing a novel tool for assessing tumor immunity and aiding in personalized treatment planning.

## Materials and Methods

### Cohort research

**Figure 1:**
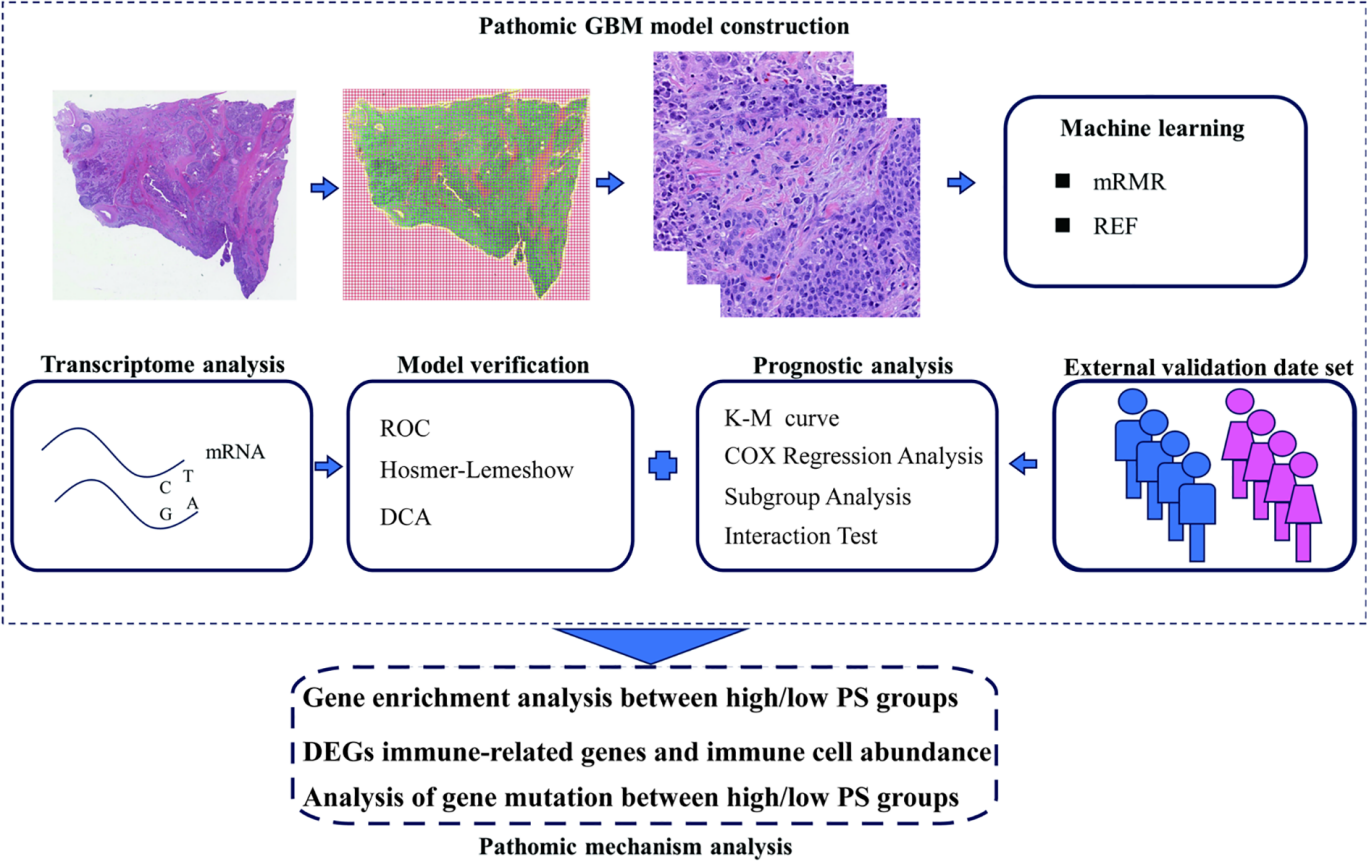
Flow chart of this study. Transcriptomic data from TCGA-HNSCC and histopathological H&E-stained images were integrated. Feature extraction and selection were performed using bioinformatics tools and GBM. External validation was conducted on a hospital cohort. GSVA and immune infiltration analyses were used to explore mechanisms of IFNG in HNSCC.

The flowchart of this experiment shows in **Figure 1**. The study utilized data from two cohorts: (1) TCGA Cohort: From the initial TCGA-HNSCC dataset containing 528 cases. The inclusion and exclusion criteria were as follows: First, 10 cases that were not primary tumors, first diagnosis, or initial treatment were excluded. Subsequently, 10 cases with missing survival data or survival time less than 30 days were removed. Four cases lacking essential clinical variables were then excluded. An additional 29 cases were excluded due to unavailable primary solid tumor samples or missing sequencing data. Finally, 204 cases with missing or substandard pathological images were excluded based on image quality assessment by experienced pathologists. This screening process ultimately yielded 271 HNSCC patients with complete clinical, genomic, and high-quality histopathological image data suitable for pathomics analysis (**Supplementary Table S1**), accessible at https://portal.gdc.cancer.gov/. (2) Hospital Cohort: For external validation, data were initially collected from 100 HNSCC patients who underwent surgical treatment in the Otolaryngology Department between December 2022 and December 2023. The inclusion criteria were: 1) pathologically confirmed HNSCC diagnosis; 2) availability of complete clinical pathology data including high-quality H&E-stained histopathological images; 3) available IFNG expression data; 4) complete follow-up information; and 5) no history of neoadjuvant chemotherapy, radiotherapy, or chemoradiotherapy prior to surgery. After applying these criteria, 71 patients were included in the final external validation cohort. The study was approved by the Human Ethics Committee of The Hospital, ensuring informed consent was obtained from all participants prior to surgery.

Patient baseline information, including age, sex, surgical treatment status, history of radiotherapy, distant metastasis, depth of infiltration, primary diagnosis, tumor location, pathological staging, and TNM staging (based on the 8th edition of the American Joint Committee on Cancer (AJCC) Cancer Staging Manual), was collected for evaluating patient prognosis.

Follow-up for surgical patients in the hospital cohort was scheduled every six months after surgery to assess survival status, including survival time, disease recurrence, and progression. Overall Survival (OS) was determined as the interval between surgery and death or the last follow-up date.

### Survival analysis

The Toil Xiantao tool (https://www.xiantao.love/products) was utilized to process RNA sequencing datasets in level 3 HTSeq Fragments Per Kilobase Million (FPKM) format from the TCGA-HNSCC project. For data visualization and survival analysis, the R packages "ggplot2" (version 3.3.3) and "survival" were employed respectively.

The "survminer" package was used for summarizing survival analysis results and visualizing them through Kaplan-Meier curves.

### Image segmentation and feature extraction

Pathological images stained with hematoxylin and eosin (H&E), were downloaded from the TCGA database (https://tcga-data.nci.nih.gov/tcga/) at magnifications of 20 × or 40 × (12). The OTSU algorithm for binary image segmentation was used to identify tissue areas (13). The 40 × images were divided into several 1024 × 1024-pixel subimages, while the 20 × images were divided into 512 × 512-pixel subimages, which were then up-sampled to 1024 × 1024 pixels. Subimages of low quality, including those with contamination, blurriness, or over 50% white space, were excluded following pathologist review. Ten subimages were randomly selected from each pathological image for further analysis (14).

Using the *"PyRadiomics"* open-source package of Python (https://pyradiomics.readthedocs.io/en/latest/), 93 original features (including first- and second-order features) based on image standardization were extracted. Wavelets (LL, LH, HL, and HH) and LoG with varying kernel sizes were among the extracted features. Through a series of transformations (squaring, square root, logarithm, exponential, gradient, LBP2D, and their combinations), a total of 1488 features were obtained. The corresponding average value of these features, calculated across the ten subimages per patient's pathological image, were used as the pathological features for subsequent data analysis (15–17).

### Feature selection and model construction

Features with zero variance and a correlation coefficient above 0.9 were removed. The "*mRMRe*" and "*caret*" packages in R were employed to identify the optimal feature subset using the mRMR and Recursive Feature Elimination (RFE) algorithms. The first 30 features were selected using the mRMR method, followed by RFE feature screening. The four pathomics features ultimately selected in this study primarily reflect the structural characteristics, heterogeneity, and spatial patterns of tissues. Specifically, GLCM (Gray Level Co-occurrence Matrix) quantifies the spatial co-occurrence relationships of gray values in images, effectively reflecting tissue structural characteristics and heterogeneity. The selected feature log_sigma_5_0_mm_3D_glcm_ClusterProminence, derived from GLCM, characterizes the kurtosis and skewness degree of gray value distribution, where elevated values indicate asymmetrically enhanced high-intensity clustering areas within tissues, typically associated with increased structural heterogeneity.

GLRLM (Gray Level Run Length Matrix) describes the distribution of run lengths where identical gray values appear consecutively in specific directions, primarily capturing the continuity and regularity of tissue texture. The selected features log_sigma_3_0_mm_3D_glrlm_RunEntropy and log_sigma_4_0_mm_3D_glrlm_RunEntropy, both derived from GLRLM, quantify the entropy of run length distribution patterns. Higher numerical values suggest greater textural complexity, disorder, and uncertainty within the image, indicating more irregular tissue texture architecture.

GLSZM (Gray Level Size Zone Matrix) measures the size distribution of connected regions composed of identical gray values, reflecting the uniformity and spatial patterns of tissue regions. The selected feature log_sigma_1_0_mm_3D_glszm_LargeAreaLowGrayLevelEmphasis, originating from GLSZM, emphasizes the presence of large, homogeneous regions with low gray level intensities. Elevated values typically correspond to areas of reduced cellular density or necrotic-like zones within the tissue.

Subsequently, a pathomics GBM prediction model was constructed using the training set to predict the gene expression based on the selected pathomics features using a gradient boosting model.

### Model evaluation

The evaluation of the GBM pathomics model was conducted using the R packages "*pROC*", "*ResourceSelection*", "*rms*", and "*rmda*". The evaluation indices were accuracy (ACC), specificity (SPE), sensitivity (SEN), positive predictive value (PPV), negative predictive value (NPV), and Brier Score. A Hosmer-Lemeshow goodness-of-fit test was used to assess the evaluation of the pathomics prediction model, along with a generated calibration curve. The comprehensive performance of the pathomics prediction model was quantified using the Brier Score. The R language "*ggpubr*" software package was utilized to visualize the difference analysis between GBM model groups. The pathomics score (PS) was combined with TCGA clinical data, and the cutoff value of the PS was calculated using the "*survminer*" software package.

### Analysis of pathological mechanisms

Gene set variation analysis (GSVA) was applied to the expression matrix of 271 HNSCC patients from TCGA to calculate the pathway enrichment scores for the Kyoto Encyclopedia of Genes and Genomes (KEGG) and Hallmark gene sets in each sample. The R package "*limma*" was utilized to analyze differences between the high and low PS groups. The first 30 pathways with the highest enrichment scores were visualized, using |t| = 1 as the significance threshold. The pathomics eigenvalues of the training set (1488 features extracted from the "PyRadiomics" package) were standardized using the z-score.

37 immune-related genes were identified (https://www.genome.jp/kegg/), and the differences in the *P*-values (*P* < 0.05) of the genes were visualized. The HNSCC samples were analyzed for gene expression using the matrix uploading feature of the CIBERSORTx database (https://cibersortx.stanford.edu/) to calculate the immune cell infiltration of each sample. An analysis of the difference in immune cell infiltration between high and low PS groups was conducted by means of a Wilcoxon rank-sum test.

Mutation data for TCGA-HNSCC patients were obtained from the TCGA data portal (https://portal.gdc.cancer.gov/), comprising 268 samples that intersected with the pathomics data. Data for somatic variants were stored in the Mutation Annotation Format (MAF), and the R package *"maftools"* was used to analyze the mutation data and visualize the top 15 mutated genes with the highest mutation frequency. The feature values in the validation set were standardized using the mean and standard deviation of the corresponding features in the training set, and the intergroup differences in clinical variables among the datasets were analyzed. The mean and standard deviation of TCGA pathological features in the training set were used to standardize the pathological features in the hospital datasets.

### Statistical analysis

Fisher's exact test or chi-squared test was employed to compare clinical features, used the "survminer" package to determine the cutoff value of PS. All categorical variables were represented as frequencies and percentages. The Wilcoxon rank-sum test for predicting gene expression level. The data underwent log2 transformation prior to analyzing the differences in IFNG expression. Use the Log-rank test for the significance test of survival rates between groups. A Cox proportional hazards regression analysis was conducted to assess the impact of one or more predictors on survival time, calculating the Hazard Ratio (HR) and 95% Confidence Interval (CI). Non-normal continuous variables were compared between groups using the Wilcoxon test. The likelihood ratio tests are used to analyze the interaction between IFNG expression and other covariates. All analyses were conducted using R software version 4.1.0 with a two-sided hypothesis test and a significance level of *P < 0.05, indicating a statistically significant difference.

The median survival time was defined as the point when the survival rate was 50%. Significance among all groups was tested using the log-rank test. Correlation analysis and univariate Cox regression were conducted to identify factors affecting OS. Subsequently, multivariate Cox regression was performed to explore whether any of these factors were independent influencing factors for OS and to assess the effects of multiple influencing factors. Furthermore, an exploratory subgroup analysis using univariate Cox regression was conducted to evaluate the impact of IFNG expression on patient prognosis across different covariates, comparing high-expression groups with low-expression groups. Interactions between IFNG expression and other covariates were analyzed using the likelihood ratio test.

## Results

### IFNG expression in HNSCC patients

We analyzed survival data from 271 HNSCC patients in the TCGA database, dividing them into two groups based on IFNG expression: a high expression group (n = 126) and a low expression group (n = 145), using a cutoff value of 0.3815 determined by Receiver Operating Characteristic (ROC) analysis (**Table 1**). Except the pathologic stage (*P* = 0.002), no statistically significant differences were observed in other factors (including age, gender, systematic_therapy, radiotherapy, perineural_invasion, margin status, primary_diagnosis, primary_tumor_site, histologic_grade) between the high and low IFNG expression groups (*P* > 0.05).

**Table 1:** Baseline clinical characteristics of 271 HNSCC patients in the TCGA cohort.

Variables	Total (n = 271)	Low(n = 145)	High (n = 126)	P*
Age, n (%)				0.784
~59	111 (41)	61 (42)	50 (40)	
60~	160 (59)	84 (58)	76 (60)	
Gender, n (%)				0.474
Female	75 (28)	37 (26)	38 (30)	
Male	196 (72)	108 (74)	88 (70)	
Systemic therapy, n (%)				0.868
NO	189 (70)	100 (69)	89 (71)	
YES	82 (30)	45 (31)	37 (29)	
Radiotherapy, n (%)				1
NO	132 (49)	71 (49)	61 (48)	
YES	139 (51)	74 (51)	65 (52)	
Perineural_invasion, n (%)				0.51
NO	106 (39)	56 (39)	50 (40)	
Unknown	66 (24)	32 (22)	34 (27)	
YES	99 (37)	57 (39)	42 (33)	
Margin_status, n (%)				0.255
Close	36 (13)	16 (11)	20 (16)	
Negative	173 (64)	99 (68)	74 (59)	
Positive	39 (14)	21 (14)	18 (14)	
Unknown	23 (8)	9 (6)	14 (11)	
Primary_diagnosis, n (%)				0.888
Keratinizing	32 (12)	17 (12)	15 (12)	
NOS	230 (85)	124 (86)	106 (84)	
Others	9 (3)	4 (3)	5 (4)	
Primary_tumor_site, n (%)				0.083
Larynx	67 (25)	43 (30)	24 (19)	
Oral Cavity	171 (63)	88 (61)	83 (66)	
Oropharynx/Hypopharynx	33 (12)	14 (10)	19 (15)	
Pathological stage, n (%)				0.002
I/II	52 (19)	21 (14)	31 (25)	
III/IV	191 (70)	115 (79)	76 (60)	
Unknown	28 (10)	9 (6)	19 (15)	
Histological grade, n (%)				0.22
G1/G2	204 (75)	114 (79)	90 (71)	
G3/G4/GX	67 (25)	31 (21)	36 (29)	

*: The continuous age variables between the two groups are compared using a two-tailed *Mann–Whitney U* test, while the other variables are analyzed using either a two-tailed *χ ^2^* test or *Fisher’s* exact test. Significance values in table: *P < 0.05.

**Figure 2:**
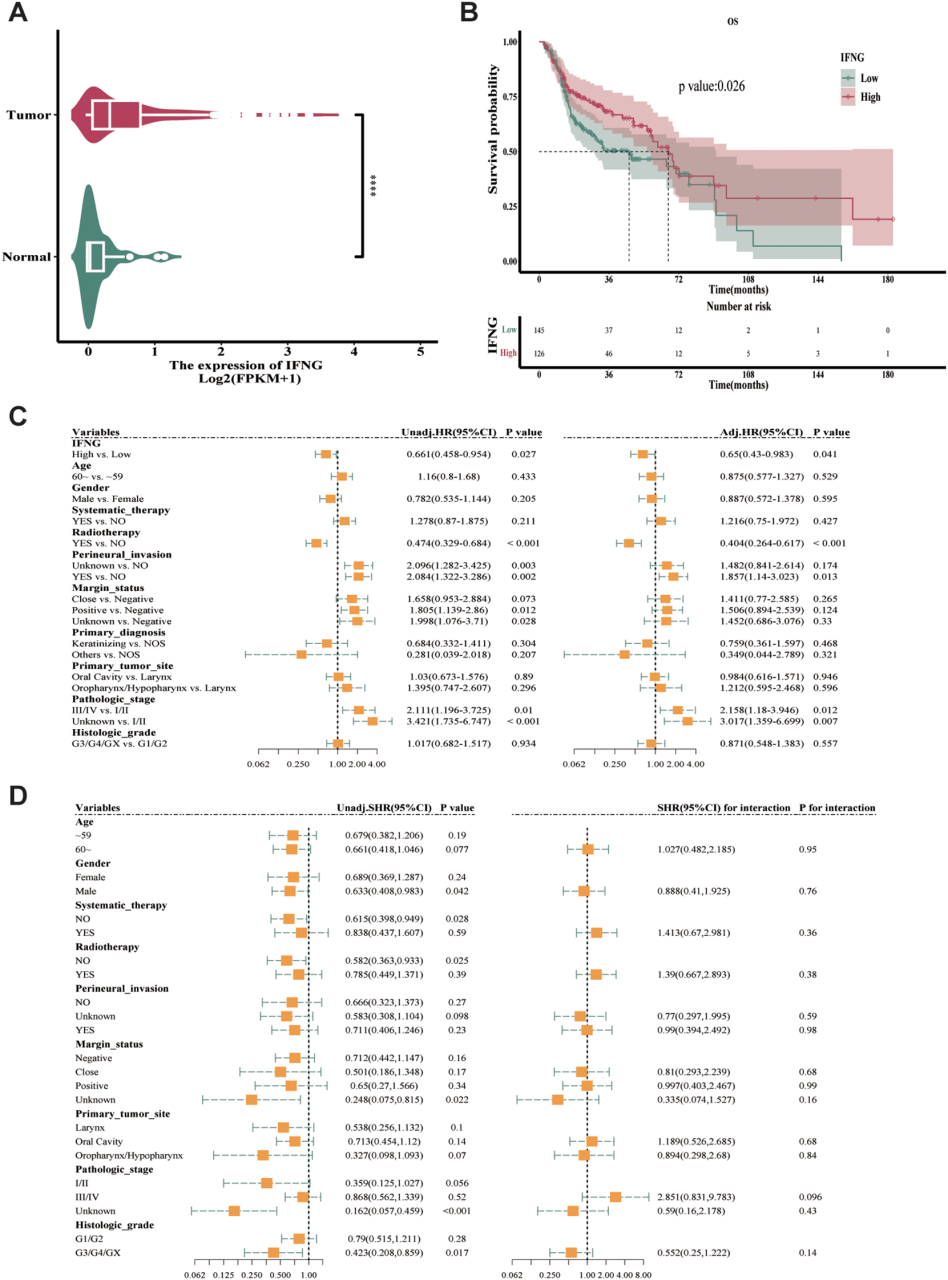
Prognostic analysis of IFNG expression. **(a)** Violin plot of IFNG expression in tumor vs. normal tissues (****P < 0.0001, Wilcoxon test). **(b)** Kaplan–Meier survival curves stratified by IFNG expression (high vs. low). Median survival time refers to the survival time corresponding to a survival rate of 50% (dash line) (log-rank test). **(c)** Forest plot of univariate and multivariate Cox regression analyses for OS. **(d)** Forest plot of subgroup analysis about IFNG expression. Significance values in figure: ****P < 0.0001.

The median survival time was 46.47 months for the low-expression group and 66.73 months for the high-expression group of IFNG (**Supplementary Table S2**). Furthermore, in survival analysis studies, KM analysis is used to estimate the survival rate and compare the survival time distribution between the high and low expression groups of IFNG. We analyzed IFNG transcriptome sequencing data from the TCGA cohort and found that IFNG levels were significantly higher in tumor tissue compared to normal tissue (****P < 0.0001) (**Figure 2a**). The analysis revealed a statistically significant median difference of 0.22066 (0.1109-0.35962) between the two groups. The KM curve analysis showed that high IFNG expression was associated with significantly improved OS (log-rank test, *P* = 0.026) (**Figure 2b**).

The Cox proportional hazards model can study the relationship between one or more study factors and the occurrence of survival outcomes. Single-factor Cox regression is used for comparative association analysis to explore factors influencing OS, while multivariate Cox regression is used to determine whether a factor is an independent predictor of OS and to examine the effects of multiple factors. When the HR value is greater than 1, the independent variable is considered a risk factor; when the HR is less than 1, it is considered a protective factor. In univariate Cox analysis, high IFNG expression was associated with reduced mortality risk (HR = 0.661, 95% CI = 0.458-0.954, *P* = 0.027). After adjusting for age, pathological stage, and margin status in multivariate Cox analysis, high IFNG expression remained an independent protective factor (HR = 0.65, 95% CI = 0.43-0.983, *P* = 0.041) (**Figure 2c**). In univariate and multivariate Cox regression analyses, high IFNG expression was associated with a significantly reduced risk of OS.

Conduct exploratory subgroup analysis using univariate Cox regression to assess the impact of IFNG (high expression group vs. low expression group) on patient prognosis across various covariate subgroups. In the subgroup analysis, in the subgroup of patients younger than 60 years old, elevated IFNG is a protective factor for OS, with a HR of 0.679, a 95% CI of 0.382-1.206, and a *P*-value of 0.19, which is not statistically significant; in the subgroup of patients aged 60 years or older, elevated IFNG is also a protective factor for OS, with a HR of 0.661, a 95% CI of 0.418-1.046, and a *P*-value of 0.077, which is not statistically significant. The interaction test yielded *P* = 0.950 (**Figure 2d**), indicating no significant heterogeneity in the prognostic effect of IFNG across age subgroups. In other words, the impact of IFNG on OS was comparable across the age subgroups.

### Pathomics analysis

#### Feature extraction, screening, and modeling

We randomly divided the data into training and validation sets with a ratio of 7:3. The training set contained 191 cases, while the validation set included 80 cases. Baseline conditions of patients in both sets were similar and comparable (*P* > 0.05) (**Supplementary Table S3**).

**Figure 3:**
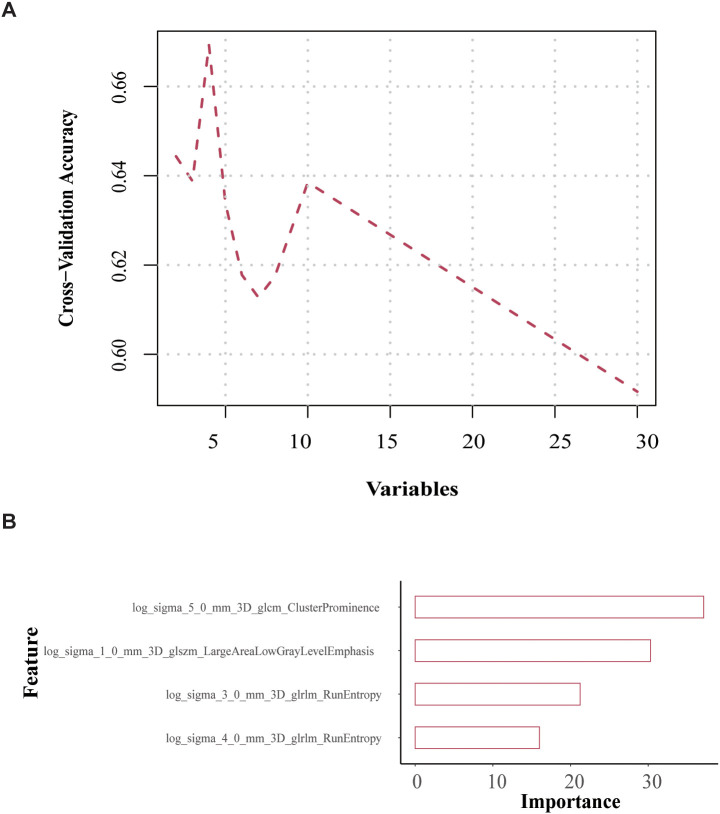
Feature selection and model construction. **(a)** Schematic of RFE for optimal feature subset selection. **(b)** Importance scores of the top 4 features in the GBM model. Features: log_sigma_5_0_mm_3D_glcm_ClusterProminence, log_sigma_1_0_mm_ 3D_glszm_Large Area Low Gray Level Emphasis, log_sigma_3_0_mm_3D_glrlm_ RunEntropy, log_sigma_4_0_mm_3D_glrlm_RunEntropy.

We used the mRMR and RFE algorithms to identify the best subset of four features, as shown in **Figure 3a**. In this investigation, we initially applied the mRMR algorithm to identify 30 features, and subsequently employed the RFE algorithm to determine the optimal 4 features, which are as follows log_sigma_5_0_mm_3D_glcm_ClusterProminence, log_sigma_1_0_mm_3D_glszm_ Large Area Low Gray Level Emphasis, log_sigma_3_0_mm_3D_glrlm_RunEntropy, log_sigma_4_0_mm_3D_glrlm_RunEntropy (**Figure 3b**).

#### Model evaluation

**Figure 4:**
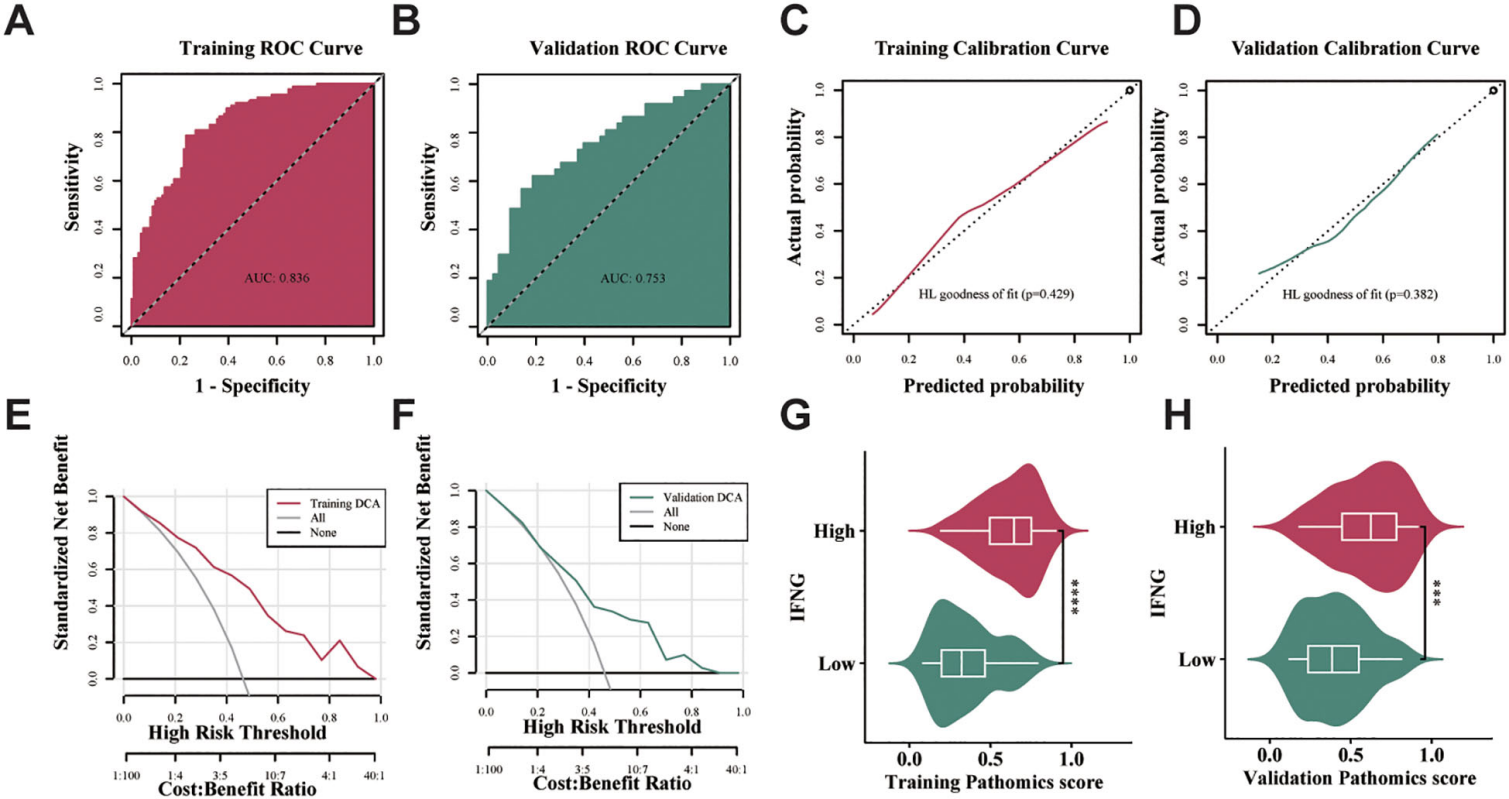
Evaluation of the efficacy of the GBM model for the validation and training sets. **(a, b)** The time independent ROC curves for OS with the GBM model in the training and validation sets. **(c, d)** Calibration curve and Hosmer–Lemeshow goodness-of-fit test of the GBM model for OS in the training and validation sets. (*P* > 0.05) **(e, f)** DCA of the training and validation sets showing clinical utility. **(g, h)** Distribution of PS between high and low IFNG expression groups ( ***P < 0.001;****P < 0.0001, Wilcoxon test).

We used ROC curves and their AUC values to evaluate the specificity and sensitivity of the machine learning models (18). The GBM model achieved an AUC of 0.836 (95% CI = 0.780-0.891) in the training set, indicating robust discriminative ability (**Figure 4a**); the GBM model achieved an AUC of 0.753 (95% CI = 0.646-0.860) in the validation set (**Figure 4b**). Based on the calibration curve and Hosmer-Lemeshow goodness-of-fit test, the pathomics prediction model predicted a true probability for high gene expression (*P* > 0.05) (**Figures 4c, d**). Additionally, we conducted Decision Curve Analysis (DCA) to illustrate the clinical benefits of the pathomics prediction model. DCA demonstrated robust clinical utility for the pathomics model in both TCGA cohorts. In the training set (**Figure 4e**), the model curve remained consistently above both the 'treat all' and 'treat none' reference lines across a broad threshold probability range from approximately 0.1 to 0.8, with peak net benefit reaching approximately 0.25-0.30 at lower threshold probabilities (0.1-0.3). In the validation set (**Figure 4f**), the model maintained clinical utility within the threshold probability range of 0.2-0.7, achieving a maximum net benefit of approximately 0.15-0.20. As shown in **Supplementary Table S4**, the threshold value of the training set was 0.474, accuracy was 0.78, sensitivity was 0.787, specificity was 0.775, and Brier Score was 0.17. The accuracy of the validation set was 0.725, sensitivity was 0.622, specificity was 0.814, and Brier Score was 0.202. The pathomics GBM model generated probability scores to predict *IFNG* gene expression levels, allowing us to examine the differences between groups with high and low *IFNG* gene expression. The PS was different between the high and low *IFNG* gene groups. The results showed that the distribution of PS in the training and validation sets was significantly different between the groups with high and low gene expression (****P < 0.0001), and the high expression group of *IFNG* exhibited significantly higher PS (**Figures 4g, h**).

#### Prognostic analysis of PS by histopathology

Building on the previous analysis of IFNG expression and its correlation with PS, the prognostic value of PS derived from histopathological data was explored. Subsequently, the continuous PS variable was divided into low and high binary groups. Baseline data tables were created for each clinical variable, with low and high PS as the groups. The GBM model, using the R package "*survminer*", predicted a cutoff value of 0.488 for PS, dividing patients into two groups: high PS expression (129 cases) and low PS expression (142 cases). There were no statistically significant differences except for margin status (*P* = 0.027) and primary tumor site (*P* = 0.009) (**Supplementary Table S5**).

**Figure 5:**
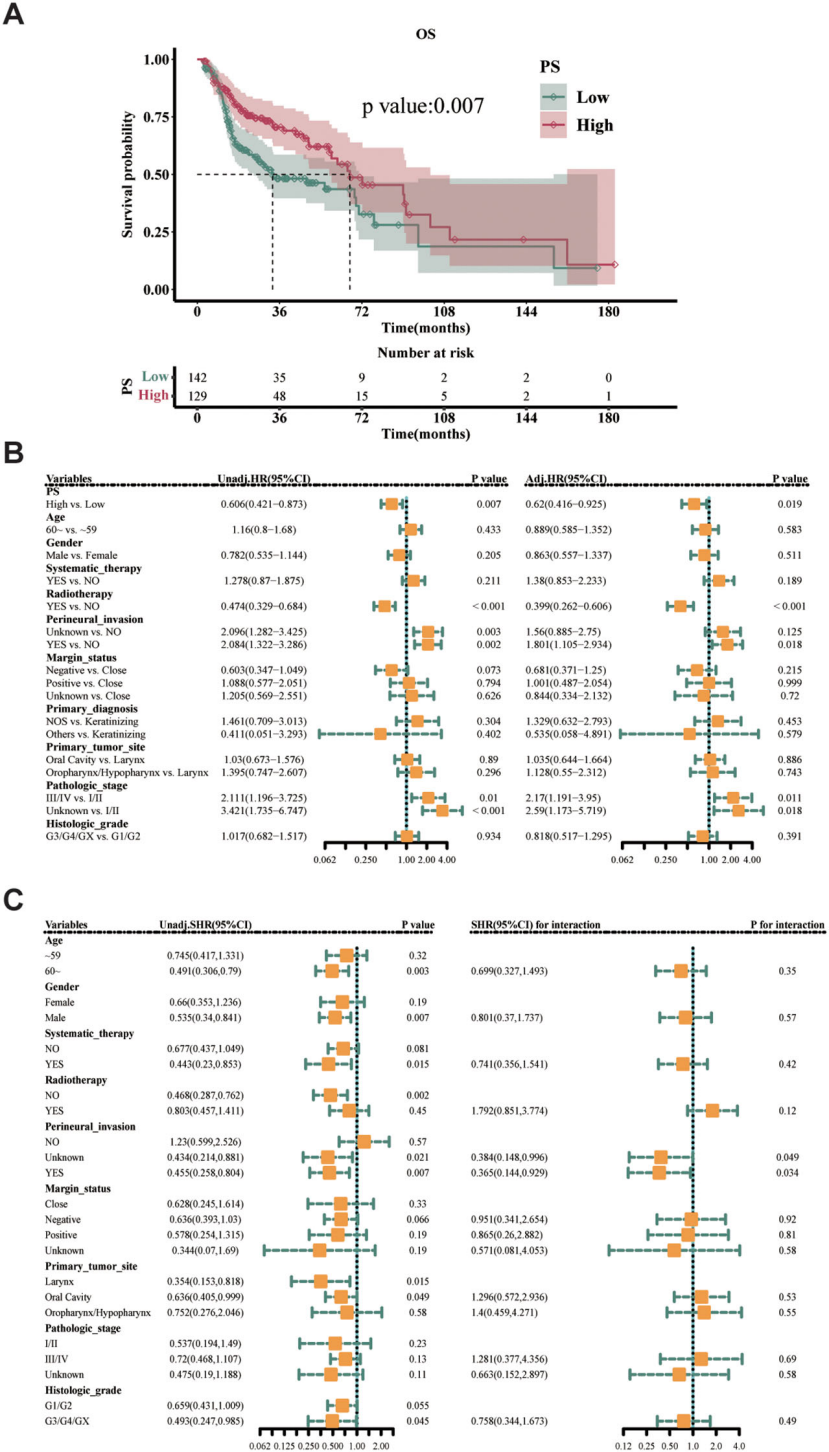
Prognostic analysis of PS expression. **(a)** The OS rate difference for high- vs. low-PS groups in the training cohort (log-rank test). **(b)** Forest plots of univariate (HR = 0.606, 95% CI = 0.421–0.873, *P* = 0.007) and multivariate (HR = 0.62, 95% CI = 0.416–0.925, *P* = 0.019) Cox regression analyses. **(c)** Subgroup analysis of PS effect on OS across age strata (interaction test).

The median survival for the high PS group was 66.73 months, significantly longer than the 3.93 months for the low PS group (*P* = 0.007, **Figure 5a**). High PS expression emerged as a protective factor for OS in both univariate (HR = 0.606, 95% CI = 0.421-0.873, *P* = 0.007) and multivariate analyses (HR = 0.62, 95% CI = 0.416-0.925, *P* = 0.019, **Figure 5b**).

Subgroup analysis showed that high PS expression was associated with a protective effect on OS in both younger (< 60 years) and older (≥ 60 years) patients, albeit non-significantly in the younger group (HR = 0.745, 95% CI = 0.417-1.331, *P* = 0.32 vs. HR = 0.491, 95% CI = 0.306-0.79, *P* = 0.003 in older patients). Notably, the interaction test revealed no significant difference in the effect of PS between age subgroups (*P* = 0.35). In other words, the effect of PS on OS was similar across both age subgroups (**Figure 5c**). As the sample size of the main variable, IFNG, in the Primary diagnosis subgroup was too small, the HR value appeared to be extreme; therefore, this variable was excluded.

#### External validation in hospital cohort

**Figure 6:**
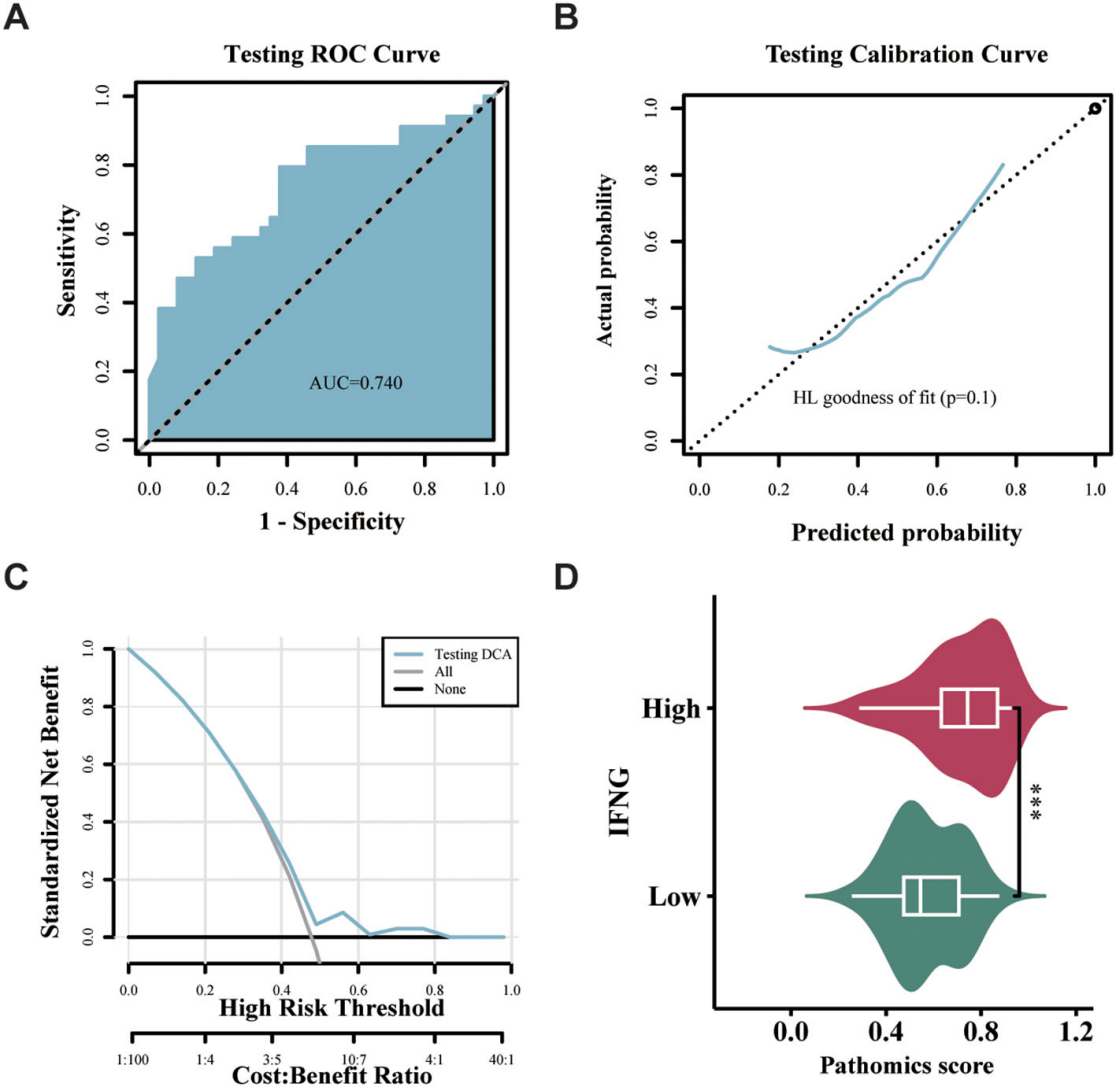
External validation of the GBM model in the hospital cohort. **(a)** The time independent ROC curves for OS with the GBM model in the hospital cohort. **(b)** Calibration curve and Hosmer–Lemeshow goodness-of-fit test of the GBM model for OS in the hospital cohort. **(c)** DCA showing clinical utility. **(d)** Distribution of PS between high and low IFNG expression groups (***P < 0.001, Wilcoxon test).

There were 71 samples in the hospital dataset with pathological images and IFNG expression data, and the median IFNG expression level of 1.5. Using the median as the threshold, the samples were divided into high and low values. We applied the same pathomic feature extraction method used in the TCGA cohort to obtain the corresponding mean and z-score normalization was performed for the pathomic feature values. As shown in the ROC curve, the AUC of the model for the hospital dataset was 0.740 (**Figure 6a**). The calibration curve and Hosmer-Lemeshow goodness-of-fit test showed that the pathomics prediction model had good consistency between the predicted probability and the true value of high IFNG expression (*P* = 0.1) (**Figure 6b**). In the external hospital validation cohort (**Figure 6c**), the pathomics model demonstrated significantly more limited clinical utility. The model curve provided net benefit only within a narrow threshold probability range of approximately 0.1-0.4, with peak net benefit reaching merely 0.05-0.10. Beyond a threshold probability of 0.4, the model curve approached or fell below the reference lines, indicating minimal clinical advantage. While the model retained some predictive value at lower threshold probabilities, the substantially reduced net benefit compared to the TCGA cohorts suggests decreased clinical applicability in this external population. As presented in **Supplementary Table S6**, the accuracy, sensitivity, specificity, and Brier Scores for the hospital validation set were 0.704, 0.794, 0.622, and 0.235, respectively. The hospital dataset showed a significant difference in PS distribution between high and low gene expression groups (***P < 0.001), with the high IFNG expression group exhibiting higher PS (**Figure 6d**).

#### Pathomics mechanism analysis

**Figure 7:**
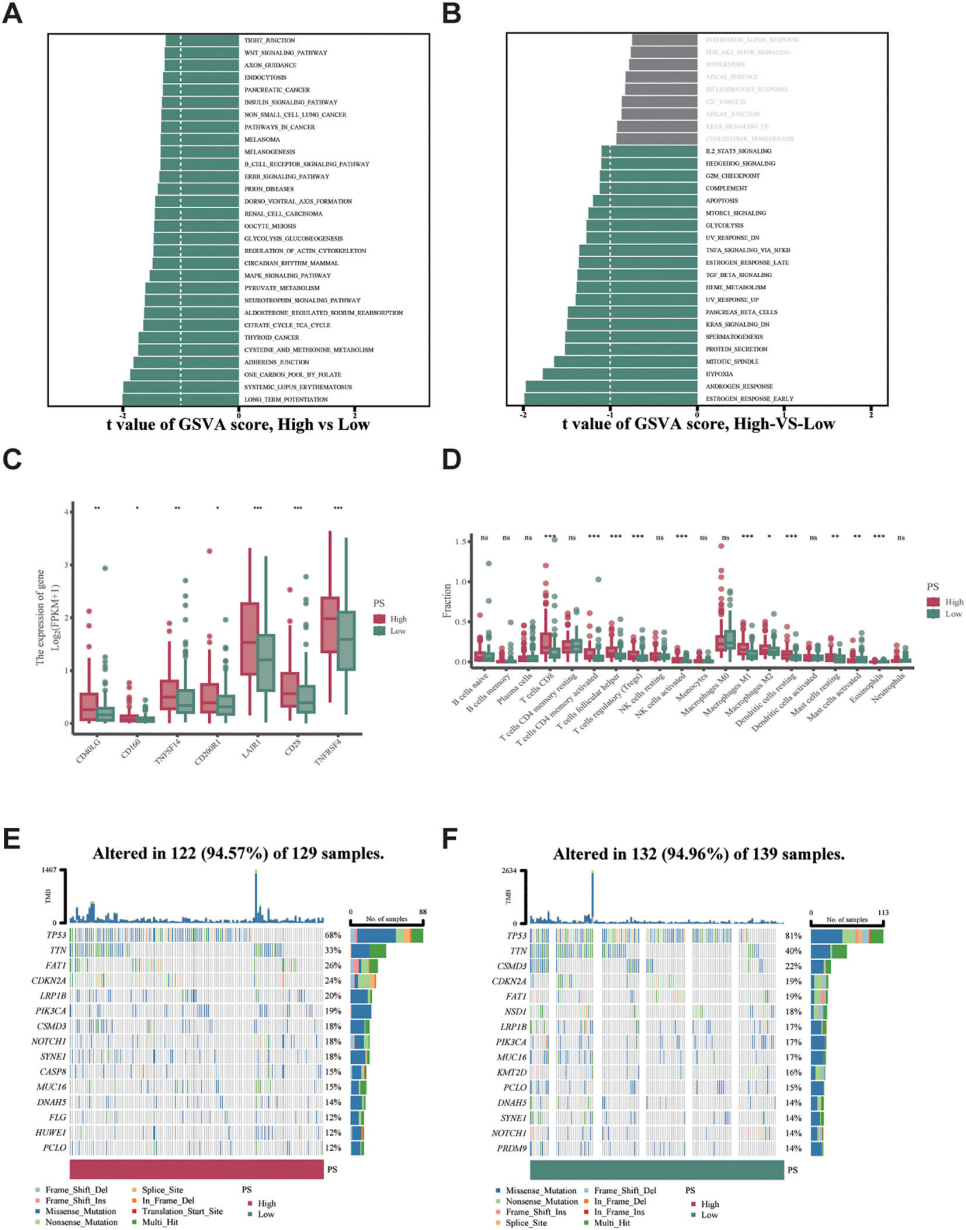
Pathomics mechanism analysis. **(a)** KEGG pathway enrichment analysis from GSVA enrichment analysis (|t| ≥ 1). **(b)** Hallmark gene set enrichment analysis (|t| ≥ 1). **(c)** Differential expression of immune-related genes (LAIR1 and TNFRSF4, ***P < 0.001). **(d)** Immune cell infiltration levels between high and low PS groups (CD8^+^ and CD4^+^ T cells, ***P < 0.001). **(e, f)** Mutation landscape in high- and low-PS groups (Visualization by maftools v2.6.05 in R 4.3.1).

We examined the enrichment of differentially expressed genes (DEGs) in high and low PS groups within HNSCC. Our findings indicated that the low PS group showed significant enrichment in the B-cell receptor signaling pathway, along with other pathways in the KEGG gene set (**Figure 7a**). In the Hallmark gene set, the differentially expressed genes in the low PS group were significantly enriched in TGF-β signaling and additional pathways (**Figure 7b**). We performed a differential analysis of immune-related genes and discovered that the expression levels of delayed activation inhibitory receptor 1 (LAIR1) and tumor necrosis factor receptor superfamily member 4 (TNFRSF4) were significantly higher in the high PS group (***P < 0.001) (**Figure 7c**). We assessed the infiltration of immune cells in the high and low PS groups. The results revealed that levels of activated memory T cells cluster of differentiation 8 (CD8) and cluster of differentiation 4 (CD4) were significantly higher in the high PS group (***P < 0.001) (**Figure 7d**).

We evaluated the relationship between the mutation spectrum of TCGA-HNSCC patients and the model-predicted PS groupings by analyzing gene mutations in the high and low PS groups using available somatic mutation data. The results showed that missense mutations were the most frequent. The mutation rate of TP53 gene was higher than 60% in both the high and low PS groups. The mutation rate of TTN gene was higher in the low PS group than in the high PS group (**Figures 7e, f**).

## Discussion

HNSCC, characterized by its high heterogeneity, poses significant challenges in clinical treatment and prognostic assessment due to its complex etiology and diverse clinical manifestations (4). In HNSCC, IFNG, as a key cytokine with antiviral, immunomodulatory, and antitumor properties, primarily functions to enhance the immune response against tumor cells by strengthening antigen presentation and promoting T-cell activation, and serves as a prognostic biomarker (19). However, the requirement of additional genetic or immunohistochemical tests limits its access to the general population. In this study, we developed a pathomics-based GBM model, a machine learning algorithm capable of handling high-dimensional feature data to predict IFNG status of HNSCC directly from histopathology images that are ubiquitously available in clinical practice, making it possible for every patient with a pathological diagnosis to receive an IFNG evaluation and guide treatment decisions and develop personalized therapeutic strategies.

Significantly, we found a correlation between high IFNG expression and improved OS in HNSCC patients (*P =* 0.026) (**Figure 2b**), indicating its potential as a prognostic biomarker. Multivariate Cox regression analysis further confirmed the independent prognostic value of high IFNG expression, with HR of 0.65 (95% CI 0.52-0.81) for reduced OS risk after adjusting for clinical variables. This result reinforces the pivotal role of IFNG in immune surveillance of cancer and its ability to control tumor progression and metastasis (20, 21). To explore the relationship between histopathological features and IFNG expression, we employed a GBM model utilizing pathomics analysis. The choice of GBM was informed by its proven effectiveness in handling high-dimensional data and complex interactions, making it well-suited for pathomics analysis (18). Despite the expected performance drop (training AUC 0.836 vs. validation 0.753), the model maintained diagnostic utility. In the external validation cohort (n = 71), the model achieved an AUC of 0.740 with acceptable calibration (Hosmer-Lemeshow test, P = 0.1) (**Figures 6a, b**). However, DCA revealed substantially reduced clinical utility in this cohort, with net benefit limited to a narrow threshold probability range compared to the TCGA datasets. The reduced net benefit observed in the hospital cohort may reflect differences in patient characteristics, imaging acquisition protocols, or sample size limitations, highlighting the importance of further prospective validation studies to optimize model performance in real-world clinical settings. While these results suggest potential generalizability, they also underscore the challenges inherent in translating pathomics models across different institutional settings.

Our finding is consistent with previous studies, which have shown that IFNG can augment the immune response against tumor cells by enhancing antigen presentation and promoting T-cell activation (22, 23). GSVA revealed significant enrichment of immune-related pathways in the low PS group, suggesting the presence of immune evasion mechanisms in these tumors. Conversely, the high PS group exhibited higher infiltration of activated memory CD8^+^ and CD4^+^ T cells, indicating a more robust anti-tumor immune response, consistent with the findings (24, 25). The GSVA pathway analysis revealed that the low PS group was enriched in immunosuppressive pathways such as TGF-β signaling, which is known to promote regulatory T-cell differentiation and inhibit CD8^+^ T-cell cytotoxicity, and B-cell receptor signaling, potentially reflecting compensatory humoral immunity in the context of impaired cellular immunity (26). These findings are consistent with the observed reduction in CD8^+^/CD4^+^ T-cell infiltration in the low PS group, suggesting a tumor microenvironment dominated by immune evasion mechanisms. Conversely, the high PS group’s association with TNFRSF4-driven T-cell activation and LAIR1-mediated regulatory feedback aligns with the enrichment of adaptive immune pathways that sustain cytotoxic T-cell function. By correlating IFNG expression with histopathological features, we were able to develop a model that not only predicts IFNG status but also provides insights into the tumor microenvironment. The identification of key immune-related genes, such as LAIR1 and TNFRSF4, whose expression levels were significantly higher in the high PS group (**Figure 7c**), adds another layer of complexity to our understanding of HNSCC immunobiology. We performed a differential analysis of immune-related genes and discovered that the expression levels of LAIR1, an inhibitory immune checkpoint molecule that suppresses T-cell activation by binding to collagen in the tumor microenvironment (27), and TNFRSF4, a co-stimulatory receptor critical for sustaining CD4^+^/CD8^+^ T-cell survival and effector function (28), were significantly higher in the high PS group (***P < 0.001). Notably, elevated LAIR1 expression may reflect a feedback mechanism to counterbalance excessive T-cell activation in immunologically active tumors, while TNFRSF4 upregulation directly aligns with enhanced T-cell proliferation and memory formation. These findings collectively suggest that the high PS group exhibits a dynamic immune microenvironment characterized by both immune activation (via TNFRSF4) and regulatory feedback (via LAIR1), which could theoretically synergize to promote durable responses, though mechanistic validation is needed. This is further supported by the significantly higher infiltration of activated memory CD8^+^ and CD4^+^ T cells in the high PS group (***P < 0.001), indicating robust adaptive immunity. The elevated expression of IFNG in HNSCC is closely linked to the infiltration and anti-tumor activity of CD8^+^ T cells. Studies have demonstrated that IFNG enhances immune cell infiltration by directly activating CD8^+^ T cells and modulating other components of the tumor microenvironment, such as CXCLs, MHC-I, and galectin-9 (Gal-9), while counteracting cancer-associated fibroblast (CAF)-mediated immunosuppression (29). Further elucidation of the IFNG-driven immunoregulatory network may reveal novel therapeutic targets to optimize immunotherapy strategies for head and neck malignancies. The model categorizes patients into distinct prognostic groups using PS values derived from image-based features, highlighting the emerging field of pathologic genomics that connects imaging phenotypes with gene expression patterns and clinical outcomes.

However, several limitations should be acknowledged. First, while our study using data of IFNG mRNA expression, mRNA levels do not always accurately reflect protein abundance, particularly for immune-related cytokines subject to post-transcriptional and translational regulation. Although the hospital cohort validation used protein-level immunohistochemical results, future studies should include comprehensive experimental evidence at the protein level, utilizing techniques such as IHC, mIHC, or ELISA to directly assess IFNG expression in HNSCC tissues. Additionally, validation studies using mouse xenograft models could further strengthen the correlation between histopathological features and IFNG protein levels, providing experimental foundation for clinical translation of histopathology-based immune biomarker prediction. Second, the markedly reduced clinical utility observed in the external hospital validation cohort, with net benefit limited to narrow threshold probability ranges, highlights significant challenges in model generalizability across different institutional settings. These performance differences likely reflect variations in patient populations, imaging acquisition protocols, and platform disparities between RNA-seq and immunohistochemical detection methods. Third, the current model requires further validation with larger sample sizes of real-world data and has not yet been developed into a deployable prediction tool or software interface for clinical implementation. Therefore, model recalibration, prospective multicenter validation studies, and the development of standardized imaging protocols are essential before widespread clinical implementation. Future research should also focus on integrating multi-omics data to enhance biological interpretability and developing user-friendly software platforms that can facilitate real-world clinical adoption of pathomics-based biomarker prediction in precision oncology practice.

## Conclusion

Our study demonstrates the prognostic value of IFNG expression in HNSCC and showcases the utility of pathomics analysis in deciphering the underlying molecular mechanisms. By integrating clinical data, IFNG expression levels, and pathological images, we leveraged the phenotypic complexity of HNSCC to explore the potential of machine learning in improving prognosis and treatment strategies. Furthermore, pathway enrichment analysis using GSVA provided deeper insights into the biological processes influencing HNSCC patient survival. Our predictive pathomics GBM model, which incorporates image-derived features, demonstrates that the tumor microenvironment captured in pathological images offers valuable prognostic information. These findings not only reinforce the prognostic significance of immune-related pathways but also suggest promising therapeutic targets for enhancing the immune response in HNSCC patients. This study provides rationale for investigating LAIR1/TNFRSF4-targeted therapies in HNSCC.

## Data Availability

The original contributions presented in the study are included in the article/**Supplementary Material**. Further inquiries can be directed to the corresponding author.
