# Supplementary material for: Pathomics-based machine learning model predicts interferon-gamma expression in head and neck squamous cell carcinoma patients
Source: Front Immunol. 2025 Nov 24;16:1652003. doi: 10.3389/fimmu.2025.1652003 (PMC12682786; doi:10.3389/fimmu.2025.1652003)
Supplement: Supplementary file 1 [file Table1.docx]

**Table S1.** TCGA database (https://portal.gdc.cancer.gov/) - HNSCC dataset inclusion and exclusion criteria

| TCGA-HNSC  （Head and Neck squamous cell carcinoma） | Exclusion | Final Sample |
| --- | --- | --- |
| Total cases | - | 528 |
| Screen out the primary, first diagnosis and initial treatment | 10 | 518 |
| Excluded missing survival data or survival time less than 30 days | 10 | 508 |
| Excluding the absence of clinical variables | 4 | 504 |
| Screen out primary solid tumors and sequencing data were available | 29 | 475 |
| Excluded samples with missing or substandard pathological images | 204 | 271 |
|  |  |  |

**Table S2.** Median survival time and KM survival curve analysis table of high and low IFNG groups

|  | records | n.max | n.start | events | rmean | se(rmean) | median | 0.95LCL | 0.95UCL |
| --- | --- | --- | --- | --- | --- | --- | --- | --- | --- |
| IFNG=Low | 145 | 145 | 145 | 71 | 56.41936 | 5.958578 | 46.46667 | 26.8 | 91.36667 |
| IFNG=High | 126 | 126 | 126 | 50 | 82.21696 | 9.449563 | 66.73333 | 55.7 | NA |

**Table S3.** Table of population clinical characteristics of 191 and 80 cases in the training and

validation sets, respectively

| **Variables** | **Total (n = 271)** | **Train (n = 191)** | **Validation (n = 80)** | ***P*** |
| --- | --- | --- | --- | --- |
| **IFNG, n (%)** | |  |  | 1 |
| Low | 145 (54) | 102 (53) | 43 (54) |  |
| High | 126 (46) | 89 (47) | 37 (46) |  |
| **Age, n (%)** |  |  |  | 0.639 |
| ~59 | 111 (41) | 76 (40) | 35 (44) |  |
| 60~ | 160 (59) | 115 (60) | 45 (56) |  |
| **Gender, n (%)** | |  |  | 1 |
| Female | 75 (28) | 53 (28) | 22 (28) |  |
| Male | 196 (72) | 138 (72) | 58 (72) |  |
| **Systematic_therapy, n (%)** | | |  | 0.433 |
| NO | 189 (70) | 130 (68) | 59 (74) |  |
| YES | 82 (30) | 61 (32) | 21 (26) |  |
| **Radiotherapy, n (%)** | |  |  | 0.5 |
| NO | 132 (49) | 90 (47) | 42 (52) |  |
| YES | 139 (51) | 101 (53) | 38 (48) |  |
| **Perineural_invasion, n (%)** | | |  | 0.454 |
| NO | 106 (39) | 75 (39) | 31 (39) |  |
| Unknown | 66 (24) | 50 (26) | 16 (20) |  |
| YES | 99 (37) | 66 (35) | 33 (41) |  |
| **Margin_status, n (%)** | |  |  | 0.097 |
| Close | 36 (13) | 26 (14) | 10 (12) |  |
| Negative | 173 (64) | 114 (60) | 59 (74) |  |
| Positive | 39 (14) | 31 (16) | 8 (10) |  |
| Unknown | 23 (8) | 20 (10) | 3 (4) |  |
| **Primary_diagnosis, n (%)** | | |  | 0.401 |
| Keratinizing | 32 (12) | 21 (11) | 11 (14) |  |
| NOS | 230 (85) | 165 (86) | 65 (81) |  |
| Others | 9 (3) | 5 (3) | 4 (5) |  |
| **Primary_tumor_site, n (%)** | | |  | 0.488 |
| Larynx | 67 (25) | 51 (27) | 16 (20) |  |
| Oral Cavity | 171 (63) | 118 (62) | 53 (66) |  |
| Oropharynx/Hypopharynx | 33 (12) | 22 (12) | 11 (14) |  |
| **Pathologic_stage, n (%)** | | |  | 0.158 |
| I/II | 52 (19) | 31 (16) | 21 (26) |  |
| III/IV | 191 (70) | 140 (73) | 51 (64) |  |
| Unknown | 28 (10) | 20 (10) | 8 (10) |  |
| **Histologic_grade, n (%)** | | |  | 0.311 |
| G1/G2 | 204 (75) | 140 (73) | 64 (80) |  |
| G3/G4/GX | 67 (25) | 51 (27) | 16 (20) |  |
| **OS, n (%)** |  |  |  | 0.552 |
| Alive | 150 (55) | 103 (54) | 47 (59) |  |
| Dead | 121 (45) | 88 (46) | 33 (41) |  |
| OS.time, Median (Q1,Q3) | 22.1 (12.83, 44.47) | 22.1 (12.62, 40.93) | 23.33 (13.22, 49.01) | 0.498 |

**Table S4.1.** Evaluation indexes of model performance (AUC area under curve, CI confidence interval, PPV positive predictive value, NPV negative value)

| **Name** | **AUC(95%CI)** | **Accuracy** | **Sensitivity** | **Specificity** | **PPV** | **NPV** |
| --- | --- | --- | --- | --- | --- | --- |
| Training set |  |  |  |  |  |  |
| IFNG_cat_PS | 0.836(0.780-0.891) | 0.780 | 0.787 | 0.775 | 0.753 | 0.806 |
| Testing set |  |  |  |  |  |  |
| IFNG_cat_PS | 0.753(0.646-0.860) | 0.725 | 0.622 | 0.775 | 0.753 | 0.714 |

**Table S5.** Baseline data of clinical variables after combining the pathomics scores with clinical data

| **Variables** | **Total (n = 271)** | **Low (n = 142)** | **High (n = 129)** | ***P*** |
| --- | --- | --- | --- | --- |
| **Age, n (%)** |  |  |  | 0.87 |
| ~59 | 111 (41) | 57 (40) | 54 (42) |  |
| 60~ | 160 (59) | 85 (60) | 75 (58) |  |
| **Gender, n (%)** | |  |  | 0.192 |
| Female | 75 (28) | 34 (24) | 41 (32) |  |
| Male | 196 (72) | 108 (76) | 88 (68) |  |
| **Systematic_therapy, n (%)** | | |  | 0.888 |
| NO | 189 (70) | 98 (69) | 91 (71) |  |
| YES | 82 (30) | 44 (31) | 38 (29) |  |
| **Radiotherapy, n (%)** | |  |  | 0.745 |
| NO | 132 (49) | 71 (50) | 61 (47) |  |
| YES | 139 (51) | 71 (50) | 68 (53) |  |
| **Perineural_invasion, n (%)** | | |  | 0.727 |
| NO | 106 (39) | 54 (38) | 52 (40) |  |
| Unknown | 66 (24) | 33 (23) | 33 (26) |  |
| YES | 99 (37) | 55 (39) | 44 (34) |  |
| **Margin_status, n (%)** | |  |  | 0.027 |
| Close | 36 (13) | 11 (8) | 25 (19) |  |
| Negative | 173 (64) | 93 (65) | 80 (62) |  |
| Positive | 39 (14) | 23 (16) | 16 (12) |  |
| Unknown | 23 (8) | 15 (11) | 8 (6) |  |
| **Primary_diagnosis, n (%)** | | |  | 0.729 |
| Keratinizing | 32 (12) | 15 (11) | 17 (13) |  |
| NOS | 230 (85) | 123 (87) | 107 (83) |  |
| Others | 9 (3) | 4 (3) | 5 (4) |  |
| **Primary_tumor_site, n (%)** | | |  | 0.009 |
| Larynx | 67 (25) | 46 (32) | 21 (16) |  |
| Oral Cavity | 171 (63) | 81 (57) | 90 (70) |  |
| Oropharynx/Hypopharynx | 33 (12) | 15 (11) | 18 (14) |  |
| **Pathologic_stage, n (%)** | | |  | 0.069 |
| I/II | 52 (19) | 20 (14) | 32 (25) |  |
| III/IV | 191 (70) | 105 (74) | 86 (67) |  |
| Unknown | 28 (10) | 17 (12) | 11 (9) |  |
| **Histologic_grade, n (%)** | | |  | 0.912 |
| G1/G2 | 204 (75) | 106 (75) | 98 (76) |  |
| G3/G4/GX | 67 (25) | 36 (25) | 31 (24) |  |

**Table S6.** Evaluation indexes of the model performance of the hospital clinical validation set

| Name | Test_auc | Test_ci | Test_acc | Test_sens | Test_spec | Test_ppv | Test_npv | Test_brierScore |
| --- | --- | --- | --- | --- | --- | --- | --- | --- |
| IFNG_cat_PS | 0.74 | 0.621-0.859 | 0.704 | 0.794 | 0.622 | 0.659 | 0.767 | 0.235 |
